# Spin injection and helicity control of surface spin photocurrent in a three dimensional topological insulator

**DOI:** 10.1038/ncomms15401

**Published:** 2017-05-22

**Authors:** Y. Q. Huang, Y. X. Song, S. M. Wang, I. A. Buyanova, W. M. Chen

**Affiliations:** 1 Department of Physics, Chemistry and Biology, Linköping University, Linköping 581 83, Sweden; 2 State Key Laboratory of Functional Materials for Informatics, CAS Center of Excellence for Superconducting Electronics, Shanghai Institute of Microsystem and Information Technology, Chinese Academy of Sciences, 865 Changning Road, Shanghai 200050, China; 3 Department of Microtechnology and Nanoscience, Chalmers University of Technology, Göteborg 412 96, Sweden

## Abstract

A three-dimensional (3D) topological insulator (TI) is a unique quantum phase of matter with exotic physical properties and promising spintronic applications. However, surface spin current in a common 3D TI remains difficult to control and the out-of-plane spin texture is largely unexplored. Here, by means of surface spin photocurrent in Bi_2_Te_3_ TI devices driven by circular polarized light, we identify the subtle effect of the spin texture of the topological surface state including the hexagonal warping term on the surface current. By exploring the out-of-plane spin texture, we demonstrate spin injection from GaAs to TI and its significant contribution to the surface current, which can be manipulated by an external magnetic field. These discoveries pave the way to not only intriguing new physics but also enriched spin functionalities by integrating TI with conventional semiconductors, such that spin-enabled optoelectronic devices may be fabricated in such hybrid structures.

## Abstract (web summary)

Surface spin current in a 3D topological insulator (TI) remains difficult to control and the out-of-plane spin texture is largely unexplored. Here, the authors identify subtle effect of the spin texture on surface photocurrent and demonstrate controlled spin injection from a semiconductor to a TI.

One of the most critical steps towards spin-functionalized electronics and optoelectronics is to generate and manipulate spin current in a desirable way^1^,^2^,^3^. In a three-dimensional (3D) topological insulator (TI), a strong spin–orbit interaction and the time-reversal symmetry result in spin-momentum locking of the surface electrons^4^,^5^,^6^, which leads to a unique spin texture unravelled by recent angle-resolved photoemission spectroscopy (ARPES) results^7^,^8^,^9^,^10^. These so-called Dirac fermions have intrigued great interest due to their exotic physical properties and promising spintronic applications^11^,^12^,^13^,^14^. By driving surface-state spin polarization out of equilibrium, an imbalanced distribution of momentum is expected to be simultaneously reached in TIs. This would generate directional and dissipationless spin current running across the surface, which has motivated intense research efforts by various approaches to achieve highly spin polarized current in TIs^14^,^15^,^16^,^17^,^18^,^19^. However, the commonly occurring metallic behaviour has unfortunately imposed a severe obstacle to controlling surface spin current^20^,^21^,^22^. McIver *et al*.^20^ recently demonstrated that, by taking advantage of optical selection rules, illumination of Bi_2_Se_3_ TI by circularly polarized light would cause a non-uniform distribution of photo-excited carriers in the **k**-space and would thereby give rise to helicity-dependent photocurrent. In TIs, this so-called circular photogalvanic effect (CPGE) should have a non-trivial dependence on the projection of the photon angular momentum with respect to the surface-state spin texture. The early study^20^ recognized the effect of incidence angle of light on helicity-dependent photocurrent. However, since most of the early studies have been limited to Bi_2_Se_3_—a prototypical TI with a rather weak hexagonal warping effect, the contribution of the out-of-plane spin texture to the photocurrent remains elusive so far. Without the involvement of all possible spin texture components, the assignment of the surface photocurrent to the spin texture is far from fully understood and complete.

In this work, the impact of the out-of-plane spin texture on surface spin current is explicitly taken into consideration and is selectively measured, by employing another 3D TI Bi_2_Te_3_ that exhibits a stronger hexagonal warping effect^8^. The magnitude and direction of the helicity-dependent surface spin photocurrent is shown to be closely related to the surface spin texture including the hexagonal warping effect, thereby providing a better understanding of the surface spin texture components and their contributions to the measured surface spin current components. We show that spin current can be photo-generated even in TI devices of the size of several millimetres, breaking the size limitation of several micrometres demonstrated so far. We further provide the first experimental proof of spin injection from an adjacent semiconductor to a TI and demonstrate control of the topological surface current by spin precession in a magnetic field, paving the way for spin transport and integration between TI/non-TI semiconductor material systems.

## Results

### Helicity-dependent photocurrent

**Figure 1:**
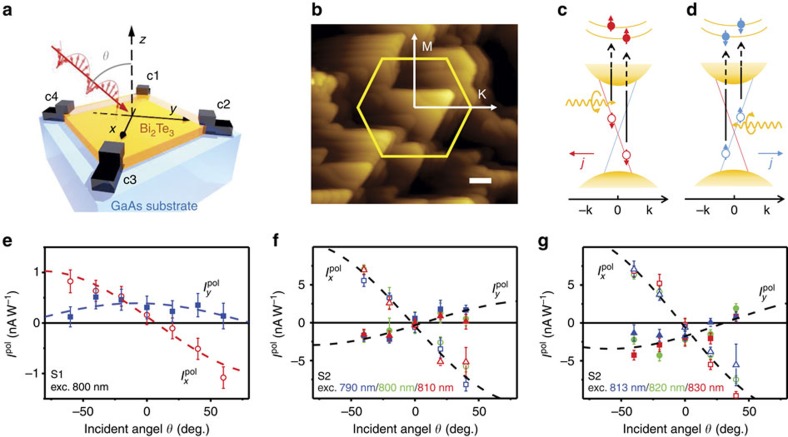
Measurement geometry and helicity dependent photocurrent from the Bi_2_Te_3_ TI devices. (**a**) A schematic picture of the device structure and measurement geometry used in this work. (**b**) AFM image of the S2 sample showing atomic layer fluctuations, together with the orientation of the surface Brillouin zone. Scale bar, 100 nm. (**c**,**d**) Schematic diagrams showing the spin-polarized surface current arising from the CPGE with the optical transitions involving the Dirac cone, illustrating the reverse of the current polarity under the light excitation of a fixed polarization but with varying incident angles *θ* from (**c**) a positive to (**d**) a negative value. (**e**–**g**) The helicity-dependent photocurrent as a function of the light incident angle measured at 5 K, obtained from device (**e**) S1 and (**f**,**g**) S2 with the specified excitation wavelengths. Both 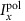 (the open symbols) and 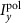 (the filled symbols) are plotted and fitted by equation (2) (the dashed lines), with the fitting parameters given in Table 1. The error bars were estimate from the statistics of 300 data points collected in steady-state measurements.

The photocurrent measurements were performed in the geometry illustrated in Fig. 1a, without external electric bias. A representative atomic force microscope (AFM) image of the sample surface and the orientation of the projected 2D Brillouin zone are shown in Fig. 1b. The excitation laser beam was directed within the *y–z* plane, where the *z* direction (that is, the surface normal) coincides with the (trigonal) *c* axis of the Bi_2_Te_3_ crystal. The photo-thermoelectric (PTE) current was intentionally suppressed by locating the laser spot at the centre of the studied TI devices where helicity-independent photocurrent along both *x* and *y* direction, which arise from bulk PTE current created by imbalanced laser heating as described in Supplementary Note 1 and Supplementary Fig. 1, was quenched. The helicity-dependent photocurrent 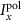
(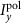) was measured by the difference between the photocurrents under *σ*^+^ and *σ*^−^ excitation to remove possible contributions from trivial effects and to single out the response of the photocurrent to photon helicity. An appreciable 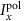 was observed whenever the incident laser beam has tilted away from the *z* direction. To clarify the origin of the photocurrent, 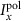 and 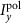 were measured as a function of the incident angle of the light and the results are shown in Fig. 1e–g for two TI devices (S1 and S2, described in Methods). We found that the helicity-dependent photocurrent has a non-trivial dependence on the incident angle such that 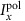 develops with increasing projection of the excitation photon **k**-vector along the *y* direction.

We note that such helicity-dependent photocurrent cannot result from the leakage current of the semi-insulating GaAs substrate, as it vanishes in control measurements of the substrate alone as shown in Supplementary Note 2 and Supplementary Fig. 2. The observed helicity-dependent photocurrent can only be explained by the CPGE through the polarization-dependent optical transitions involving the surface helical states, as shown in Fig. 1c,d. In our *n*-type Bi_2_Te_3_, circularly polarized light creates electrons in the higher lying spin-degenerate bulk states and leaves the corresponding holes in the helical state 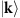. The helicity-dependent photocurrent is primarily carried by the holes in the Dirac cone, while the electrons created in the bulk states have group velocities that tend to cancel. As given in Supplementary Notes 3 and 4 and Supplementary Fig. 3, the photocurrent density is determined by the helicity-dependent part of the transition matrix element that couples the initial helical states with the bulk states^9^,^23^


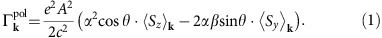


Here *α* and *β* describe the non-vanishing velocity matrix elements imposed by the symmetry consideration. 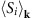 represents the pseudospin projection in the *i*th direction associated with the helical state 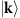. Equation (1) suggests that the optical transition induced by circularly polarized light takes place asymmetrically in the *k*-space with the transition probability proportional to the surface spin texture. The 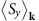 term vanishes at 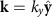, and therefore does not contribute to 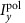 measured along the *y* direction. However, it would create a difference in the absorption between 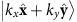 and 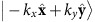 and thus an imbalance of the photo-carriers in the way that the current is created only along the *x* direction with a value proportion to sin *θ* that vanishes at *θ*=0°. At the same time, another current component is predicted to be associated with the out-of-plane spin texture 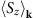, which has been shown by theory and ARPES experiments to be important in Bi_2_Te_3_ due to an enhanced hexagonal warping effect^8^. This current is expected to follow an angular dependence described by cos *θ* that does not vanish at *θ*=0°.

Based on the above theoretical analyses, the measured angular dependence of 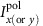 can be fitted by the expression


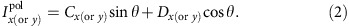


Here 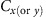 and 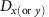 describe the surface current components associated with the in-plane and out-of-plane spin texture, respectively. Ideally, *C*_*y*_=0 in our experimental geometry as discussed above. In practice, however, it could gain a small value if the experimental geometry was not perfect, for example, the laser beam tilted away from the *y–z* plane or/and the *x* and *y* directions are not perfectly orthogonal. As shown in Fig. 1e–g, good agreement is obtained between equation (2) and the experimental results with the fitting parameters (given in Table 1) obtained from the best fit. From these results, the following conclusions can be made. First, the contribution to 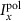 from the in-plane spin texture is significantly larger than that from the out-of-plane spin texture 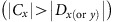, which is consistent with the ARPES results and theory. Second, 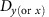 has a non-zero value under the optical excitation below the bandgap of GaAs 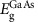, providing a direct proof for the out-of-plane spin texture and the hexagonal warping effect that was previously overlooked in the spin current measurements. Third, 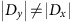 under the optical excitation below 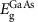, which reflects the anisotropic spin texture in the **k**-space described in Supplementary Note 3. Furthermore, 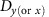 exhibits a strong dependence on the excitation wavelength due to spin injection from GaAs with an out-of-plane spin orientation (to be described below).

We should note that our TI films are thin enough to allow light absorption by the bottom surface. The measured helicity-dependent surface spin photocurrent should therefore be a result of combined contributions from both top and bottom surfaces. As the two surfaces exhibit opposite signs in the spin-momentum locking leading to opposite surface photocurrent, the observation of a non-vanishing net surface spin photocurrent is facilitated by a larger contribution from the top surface than the bottom surface due to effects like light absorption by the bulk of the Bi_2_Te_3_ films as described in Supplementary Notes 3 and 5, such that the contributions from the two surfaces are not completely cancelled out.

### Spin injection from GaAs to TI

Our conclusion on spin injection from GaAs to TI is based on the following consistent experimental findings from our systematic and correlative studies.

First, penetration of the excitation light through the Bi_2_Te_3_ film that can reach the GaAs substrate. The prerequisite for photo-generation of carriers in the GaAs substrate is that the excitation light was capable of penetrating the Bi_2_Te_3_ films and reaching the substrate. This is confirmed by the results from optical transmission measurements that showed that a noticeable portion (on the order of a few per cent) of the excitation light with a photon energy (*E*_exc_) near the GaAs bandgap 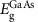 could penetrate the Bi_2_Te_3_ film and reached the GaAs substrate, as described in Supplementary Note 5 and Supplementary Fig. 4.

**Figure 2:**
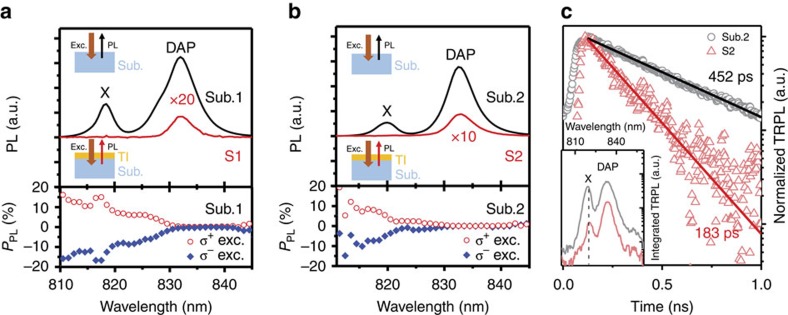
Cw- and TR-PL from the GaAs substrate. (**a**,**b**) cw-PL (the solid black curves) and PL circular polarization *P*_PL_ (the red and blue symbols) spectra from the bare GaAs substrates Sub.1 and Sub.2. The solid red curves are the PL spectra of the GaAs substrate from the S1 and S2 samples, respectively, under the optical excitation through the Bi_2_Te_3_ film. The DAP PL emission from the S1 and S2 sample is unpolarized, similar to that in the Sub.1 and Sub.2 samples, and therefore their polarization curves are not shown for clarity. The excitation photon energy was above GaAs bandgap (at 750 nm) and excitation power was kept at 60 mW. The resulting PL was measured in a back-scattering geometry as illustrated by the measurement schemes. All spectra were taken at 5 K. (**c**) TR-PL decay curves of the excitons X from the GaAs substrate, obtained at 5 K from the S2 sample (the red open triangles) and the Sample Sub.2 (the grey open circles) under the pulsed optical excitation with photon energy above the GaAs bandgap (at 750 nm). The solid lines are the fitting curves with the specified decay times. The insert shows the PL spectra from the S2 sample and the Sub.2 sample, integrated over the first 20 ps after the laser pulse. The excitation power was adjusted between the S2 sample and the Sub.2 sample, guided by the optical transmission data, such that the GaAs substrate was excited with the same optical excitation density in both cases.

Second, generation of electrons and holes in the GaAs substrate by the penetrating light. An unambiguous proof for carrier generation in the GaAs substrate by the penetrating excitation light with 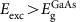 is provided by the appearance of the characteristic photoluminescence (PL) emission arising from the well-known donor–acceptor pair (DAP) recombination in GaAs, see the red curves in Fig. 2a,b, revealed in our steady-state-PL (cw-PL) experiments.

Third, spin polarization of the photo-generated electrons in the GaAs substrates by the penetrating circularly polarized light. A definite proof for electron spin generation in the GaAs substrates by the circularly polarized light is provided by a sizable circular polarization degree *P*_PL_ of ∼10% observed from the band-edge excitons (denoted as X in Fig. 2a,b) in the bare GaAs substrates (Samples Substrate 1 and Substrate 2 or in short Sub.1 and Sub.2) under the normal incidence of circularly polarized light. As *P*_PL_ of the excitons with spin-depolarized holes is governed by the exciton electron spin polarization 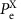 and X is contributed by both heavy-hole and light-hole excitons with a 3:1 intensity ratio but with opposite *P*_PL_ for a given 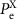 (refs 24, 25), the corresponding |
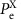
| in the exciton ground state should be twice as large as |*P*_PL_|, that is, |
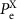
|∼20%. Taking into account possible spin losses during capture of conduction band (CB) electrons and valence-band holes in forming the excitons, we can therefore conclude that the spin polarization degree 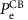 of the photo-generated CB electrons in the GaAs substrates must be >20% but <50% (50% is the theoretical maximum spin polarization degree, 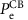, that CB electrons can acquire under the 100% circularly polarized optical excitation with the photon energy above the GaAs bandgap but without involving the spin-orbit split-off valence band state. Considering structure inversion asymmetry at the surface for the bare GaAs substrates and the Bi_2_Te_3_/GaAs interface for Samples S1 and S2, electron spin relaxation could be more severe due to the Rashba effect than that in the bulk. However, such spin depolarization effect could be reduced to some extent by the expected shorter lifetime of free carriers due to non-radiative carrier combination via possible surface and interface defects as well as spin injection in Samples S1 and S2, since 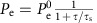. Here 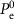 and *P*_e_ are the electron spin polarization degrees before and after undergoing spin relaxation, *τ* and *τ*_s_ are the lifetime and the spin relaxation time, respectively. Nevertheless, the maximum spin polarization achievable for the CB electrons under the circularly polarized optical excitation is most likely markedly below 50%.) The observed vanishing *P*_PL_ for the distant DAP recombination in GaAs is expected from an extremely long lifetime of the electrons (holes) localized at the donors (acceptors) that is much longer than its typical spin relaxation time as discussed in Supplementary Note 6 and Supplementary Fig. 5. (The absence of the X emission in the cw-PL spectra from Samples S1 and S2 under the optical excitation of the substrate through the TI film can be explained by the lower density of the excitation light reaching the GaAs substrates due to light reflection and absorption by the TI films and, more importantly, the shorter exciton lifetimes due to the spin injection from GaAs to the TI that was confirmed experimentally as will be described below.)

**Figure 3:**
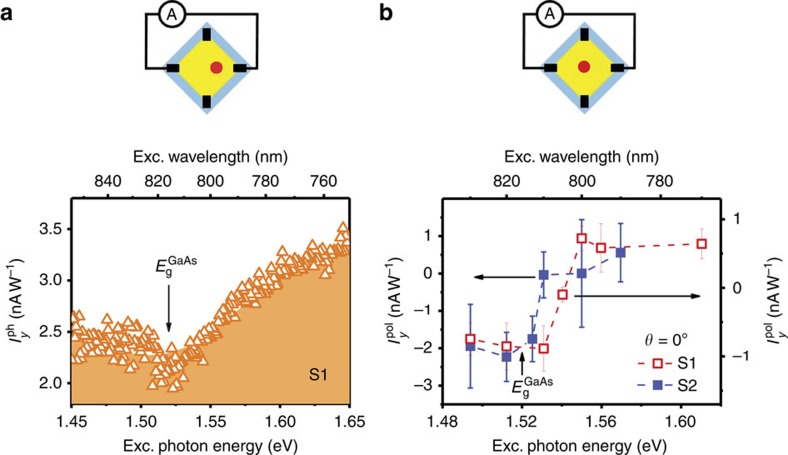
Carrier and spin injection from GaAs to TI. (**a**) PTE current 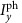 in the S1 sample, generated intentionally under an imbalanced excitation condition. The current rise observed when the photon energy is tuned above the bandgap energy of GaAs (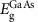), with the onset around 1.52 eV, is a result of carrier injection from the GaAs substrate to TI. (**b**) Helicity-dependent photocurrent 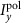 as a function of excitation photon energy at the normal incidence measured at 5 K from both S1 and S2 samples. The error bars were estimated from the statistics of 300 data points collected in steady-state measurements.

Fourth, significant injection of photo-excited carriers from GaAs to TI. The evidence for injection of photo-generated carriers from GaAs to TI is directly provided by a substantial increase of PTE current 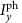 when 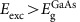. (For the measurements of 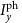, an imbalanced geometry was employed to provide a non-vanishing 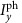 that can directly monitor injection of hot carriers from GaAs and its relative contribution as compared with the PTE current component arising from the photoexcitation of the Bi_2_Te_3_ film alone.) Carrier injection gives rise to an increase of ∼40% in 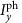 as compared with that observed with 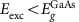, as shown in Fig. 3a. As the contribution to 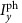 from photo-generation of hot electrons within Bi_2_Te_3_ itself should be smoothly varying with 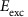 near 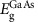, which is well above the Bi_2_Te_3_ bandgap energy 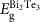, the observed abrupt increase of 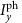 when 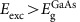 must arise from a different source of hot electrons other than Bi_2_Te_3_. The possibility that the observed 40% increase in 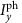 is merely due to reabsorption of the PL emission light from the GaAs substrate by the TI film can be safely excluded here as the DAP intensity from the substrate was experimentally measured to be at least six orders of magnitude weaker than the laser light. This leaves the injection of photo-generated electrons from GaAs to TI, driven by a large bandgap mismatch between them (
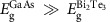
), as the only plausible explanation for the observed sharp increase of 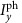 in Bi_2_Te_3_.

To further confirm carrier injection from GaAs to Bi_2_Te_3_, we have conducted time-resolved PL (TR-PL) experiments to examine carrier/exciton lifetime in GaAs that could be affected by the injection process. As band-edge excitons in GaAs are formed by binding photo-generated electron–hole pairs and free excitons additionally have a chance to diffuse to the GaAs/TI interface, thereby contributing to carrier injection, the exciton emission X was closely monitored in our TR-PL studies. It is clearly seen from the results shown in Fig. 2c that the exciton lifetime of the GaAs substrate is significantly shorter in the Bi_2_Te_3_/GaAs structure (for example, Sample S2) than that in the reference sample of the bare GaAs substrate (for example, Sample Sub.2). Besides the radiative and non-radiative recombination processes common to GaAs in both the Bi_2_Te_3_/GaAs structure and the bare GaAs substrate, a major difference between the two structures that can affect the GaAs exciton lifetime is the presence of an additional loss channel for carriers/excitons in GaAs due to their injection to Bi_2_Te_3_ in the former structure. This finding thus provides further evidence for efficient carrier/exciton injection that tends to deplete the carriers/excitons in GaAs at and near the TI/GaAs interface.

Fifth, effect of the spin orientation of the electrons injected from the GaAs substrate on the surface spin photocurrent in Bi_2_Te_3_. The effect of spin injection from GaAs into Bi_2_Te_3_ was examined under circularly polarized light excitation at normal incidence and in a balanced geometry. The choice of this excitation condition was to ensure that the CPGE induced by the in-plane spin texture within Bi_2_Te_3_ is minimized (see equations (1) and (2)), as well as spin generation and injection from GaAs are most efficient when the circularly polarized light propagates along the direction normal to the GaAs/Bi_2_Te_3_ interface (that is, the spin states remain well defined with the quantization axis along the surface normal). The effect of spin injection was uncovered through the observation of a sharp, non-trivial change of helicity-dependent photocurrent 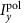 when *E*_exc_ was tuned across 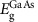, as shown in Fig. 3b. 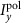 was chosen here because it is created only through the out-of-plane spin texture (with the largest matrix element) and thus is most sensitive to the injection of carriers from GaAs with the out-of-plane spin orientation. As the PL intensity from the GaAs substrates is negligibly low as compared to the laser light, we can safely rule out reabsorption of the PL emission from GaAs as a possible explanation for the experimental observation. The strong variation of 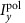 observed across 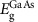, especially the change in 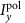 parity, is neither expected from a Fermi level shift upon carrier injection, as shown in Supplementary Note 4.

**Figure 4:**
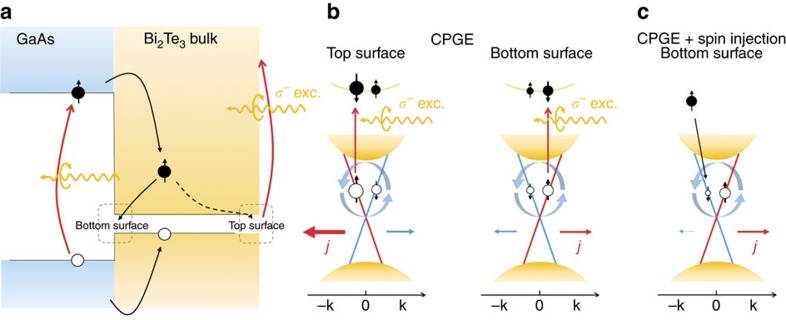
Model of the spin injection-induced modification of the surface spin photocurrent. (**a**) A simplified energy diagram by lining up the vacuum levels of GaAs and Bi_2_Te_3_ without considering any charge transfer process between the two materials. (**b**) A schematic diagram showing the preferred generation of spin-up holes in the Dirac cone and the resulting spin-polarized photocurrent on the top and bottom surface, arising from the CPGE with the direct optical transitions in Bi_2_Te_3_ under the *σ*^−^ polarized excitation. (**c**) Illustration of selective annihilation of the spin-down holes on the bottom surface with the spin-up electrons injected from the GaAs substrate under the *σ*^−^ polarized excitation, which enhances the bottom-surface spin photocurrent and competes with the spin photocurrent on the top surface generated by the CPGE. Effect of the spin injection on the top-surface spin current is expected to be negligible due to an extremely short spin diffusion length in the bulk Bi_2_Te_3_, and is therefore not shown here.

The observed abrupt change of 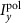 accompanied by a switching of the 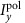 direction can only be explained by the spin injection process from GaAs, as illustrated in Fig. 4. It is well known from optical orientation that above-bandgap optical excitation of GaAs (without involving the spin-orbit split-off holes) with a given circular polarization, say *σ*^−^, predominately generates spin-up electrons and spin-down holes^24^,^25^. As holes experience stronger spin relaxation in GaAs and slower injection due to a lower mobility as compared with electrons, it is reasonable to view that spin injection is mainly associated with electrons as illustrated in Fig. 4a. Under the same polarized excitation, the direct optical absorption process in TI is determined by the helicity-independent transition matrix element 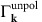 and the helicity-dependent transition matrix element 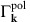 as described in Supplementary Note 3. The former creates both spin-down and spin-up holes in the helical states, whereas the latter preferably generates spin-up holes. If the momentum (spin) relaxation does not completely destroy the hole spin polarization, the spin-up holes are in majority and determine the polarity of the surface spin current following the surface spin texture (see Fig. 4b). Although the polarity of the surface spin current is expected to be opposite between the top and bottom surfaces, they do not completely cancel out due to the imbalance in the excitation light intensity between the two surfaces. The net surface current should follow the direction on the top surface due to its larger contribution. The situation changes upon injection of spin-polarized electrons from the GaAs substrate. Such spin injection is expected to predominantly affect the bottom surface that is interfacing with the GaAs substrate, due to an extremely short spin diffusion length in the bulk Bi_2_Te_3_. The selective annihilation of the spin-down surface holes with the spin-up electrons injected from GaAs thus tends to reduce the number of the spin-down surface holes on the bottom, as shown in Fig. 4c, leading to an increase of the spin photocurrent on the bottom surface. Since the bottom-surface spin photocurrent runs in the opposite direction as the top-surface spin photocurrent, a decrease in the net spin photocurrent is expected and eventually even a change in polarity upon increasing spin injection could occur. This was indeed experimentally observed in our TI, as shown in Fig. 3b. It is interesting to note that the change in the surface spin current due to the spin injection from GaAs to TI amounts to >100%, much higher than the increase of 40% in the helicity-independent PTE current, underlining the effectiveness of the spin injection on controlling the surface spin current.

### Control of surface spin current by a magnetic field

**Figure 5:**
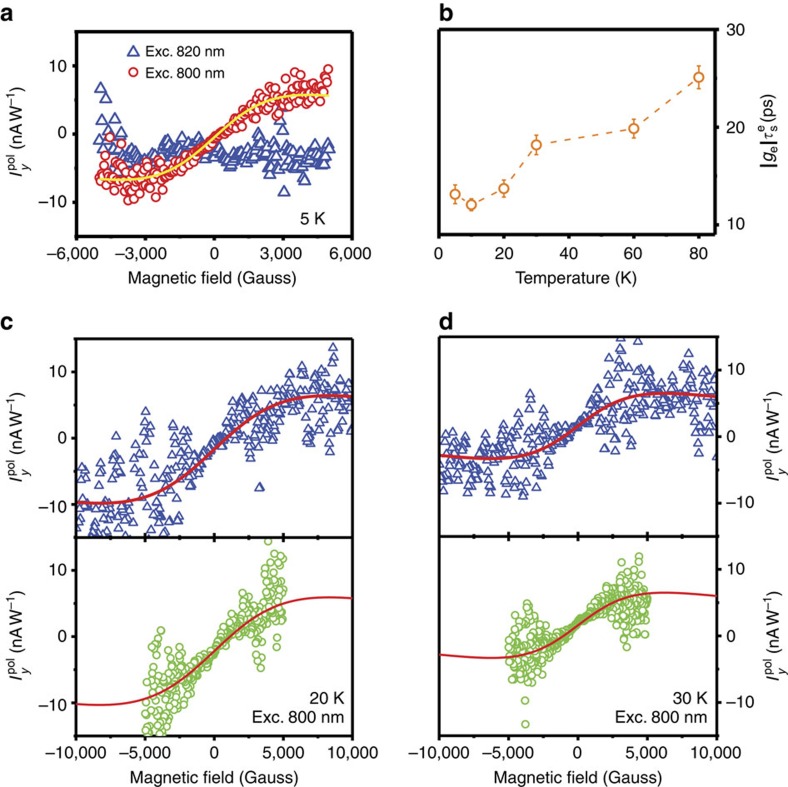
Surface spin photocurrent under spin precession of the injected electrons from GaAs. (**a**,**c**,**d**) Magnetic-field dependence of 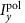 measured from the S2 device at 5, 20 and 30 K in an applied magnetic field along the *y* direction, under the excitation with the photon energy above (800 nm) and below (820 nm) the GaAs bandgap to highlight the effect of the spin injection. The data obtained over both wide- and narrow-field ranges are shown in **c**,**d**, with the latter being measured with a finer field step. The symbols denote the experimental data and the solid lines are the fitting curves based on equation (3). (**b**) Temperature dependence of the 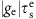 values obtained by fitting the field dependence of 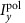. The excitation power was kept constant at 40 mW for all measurements. The error bars were received from the least-square-fitting procedure of the field-dependent response of 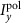 using equation (3) for different temperatures.

The uncovered spin injection from GaAs provides an unprecedented opportunity to manipulate spin current in the TI by controlling electron spins in GaAs before they are injected into the TI. The feasibility of such approach is demonstrated in Fig. 5a,c,d, where 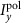 is controlled by a magnetic field along the *y* axis that is perpendicular to the spin axis (along the *z* axis) of the CB electrons generated in GaAs at normal incidence of circularly polarized light. We found that, with 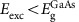, 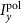 show no field dependence. This is consistent with the general belief of the fast spin relaxation and dephasing process within the TI. However, once *E*_exc_ was tuned above 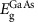, an antisymmetrical field dependence for 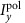 is observed as shown in Fig. 5a,c,d.

The observed field dependence of 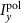 can be understood by considering that the electron spins in GaAs, originally oriented along the *z* direction, undergo spin precession in the *x–z* plane around the magnetic field direction (*y*) with a Larmor frequency 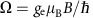. Here, *g*_e_ is the transverse electron *g*-factor of GaAs and *μ*_B_ is the Bohr magneton. Such precession can be interrupted by events of electron spin relaxation and electron losses (due to injection, trapping, recombination and so on), which is commonly described by an effective electron spin lifetime 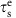. It is a dynamic balance between these two fast processes that results in a finite value in an averaged non-equilibrium spin projection along the *x* direction 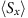, which contributes to the surface current along the *y* direction (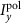) after the electrons are injected to the TI. The magnitude of 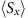 and thus 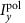 are determined by the relative values of 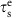 and the spin precession period (1/Ω), which should be field dependent as described in detail in Supplementary Note 7 and Supplementary Fig. 6. If the field direction is reversed, the electron spin precession direction in GaAs also reverses leading to an antisymmetrical behaviour of 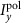 as seen in Fig. 5a,c,d. The field dependence of 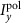 can be described by the following relation^26^,


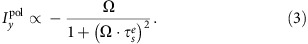


We show that the experimental results can be satisfactorily fitted by equation (3), shown by the solid lines in Fig. 5a,c,d, from which 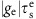 values can be extracted and are plotted in Fig. 5b as a function of temperature. By using the well-known *g*-factor *g*_e_=−0.44 for GaAs, we found that 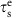 increases with increasing temperature and has a value around 60 ps at 80 K. The deduced values fall within the range of 20–150 ps typically reported for electron spin lifetime in GaAs bulk and quantum well structures^27^,^28^,^29^. The observed temperature dependence of the spin lifetime is also consistent with what is expected from the D'yakonov–Perel' spin relaxation mechanism^29^,^30^. (We point out that 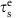 deduced from the fitting is an effective averaged value, as the experimental results were obtained from ensemble electrons in GaAs with a distribution of 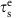 depending on their proximity to the Bi_2_Te_3_/GaAs interface.) The fact that the surface spin current of the TI can be significantly altered and controlled by the spin injection from GaAs suggests that the TI can greatly benefit from the adjacent GaAs in such hetero or hybrid structures where one can exploit GaAs for its longer electron spin lifetime, improved performance at elevated temperatures and control of spin current in TI.

## Methods

### Samples

The investigated devices (denoted by S1 and S2) were made from Bi_2_Te_3_ thin films prepared by molecular beam epitaxy on a semi-insulating GaAs (111)B and GaAs (100) 2° off-cut substrate, respectively. The film thickness was controlled to be 50 nm for both samples, which was sufficiently large to exclude any noticeable interaction between the top and bottom surfaces of Bi_2_Te_3_, while it was still thin enough to allow light absorption in the GaAs substrate close to the interface with the TI that was essential to spin injection. The surface height fluctuations were about 5 and 8.4 nm for the Samples S1 and S2, respectively, estimated from a quantitative analysis of the statistical distributions based on their AFM images as described in Supplementary Note 8 and Supplementary Fig. 7. The observation of non-vanishing surface spin photocurrent over a long distance on the order of mm in these two samples shows that the surface spin effect is rather tolerant to the TI film thickness variations (up to 10–15% known in our samples). After removal from the growth chamber, both Bi_2_Te_3_ films were cleaved into square samples of 5 mm × 5 mm with the edges along the <110> direction of the respective substrates for S1 and S2. X-ray diffraction confirmed that the growth only took place along the *c* axis of the Bi_2_Te_3_ crystals regardless of the substrate crystallographic orientations and the resulting films were highly uniform with good crystallinity, as shown in Supplementary Fig. 8 and Supplementary Note 8. Within the films, the orientations of the crystal axes could directly be distinguished from the AFM images showing atomic layer fluctuations with the cleaved edges following the trigonal crystal structure. As can be seen in Fig. 1b, even though domains with 60° rotation exist, a large area of the films consists of well aligned subcrystalline domains such that any anisotropic effect is not expected to average out. The orientation of the 2D Brillouin zone projected onto the sample surface was then determined with respect to this major crystal orientation as indicated in Fig. 1b. By correlating the AFM result with the substrate orientation, we found that the mirror plane 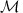 defining the *C*_*v*_ symmetry of Bi_2_Te_3_ lies along the cleaved edge of both samples. The devices were fabricated with direct contacts made from indium solder on each corner of the Bi_2_Te_3_ films as illustrated in Fig. 1a, and the *x* and *y* directions of the current are defined along the diagonal contacts. We note that for both devices the out-of-plane spin texture induced by the hexagonal warping effect does not vanish along the *x* and *y* directions. At room temperature, the resistivity of the films was measured to be in the typical range for metals (1.8–1.9 × 10^−5^ Ω·m), following the standard Van der Pauw method. With decreasing temperature, the resistance of the S1 device measured between the electrode c1 and c3 rapidly decreases until a saturation is reached below 50 K as presented in Supplementary Note 1. This indicates a metallic behaviour, commonly seen in as-grown Bi_2_Te_3_ films with the Fermi level lying above the CB edge. The studied samples show exceptionally good electrical properties with Hall mobilities of 7,010 and 5,450 cm^2^ V^−1^ s^−1^ at 5 K for Samples S1 and S2, respectively.

The bare substrates used in the growth of the TI film in Samples S1 and S2, namely the semi-insulating GaAs (111)B and GaAs (100) 2° off-cut substrate that are denoted as Sub.1 and Sub.2, respectively, were also studied as the reference samples.

### Experimental techniques

Photocurrent was measured independently for both *x* and *y* directions without electric bias, under optical excitation with a wavelength-tunable, cw Ti-sapphire laser. The excitation light was modulated either in intensity by a mechanic chopper to generate helicity-independent photocurrent or in helicity by using a broadband electro-optic amplitude modulator to generate helicity-dependent photocurrent. Both helicity-independent and helicity-dependent photocurrent could be separately and selectively registered with the standard lock-in technique as described in Supplementary Note 9 and Supplementary Fig. 9. In the helicity-dependent photocurrent measurements, electro-optic amplitude modulator introduced a periodic variation of the laser polarization with each cycle starting from *σ*^+^ (left circularly polarized) to *σ*^*y*^ (linearly polarized along the *y* direction), then to *σ*^−^ (right circularly polarized) and finally back to *σ*^+^ via *σ*^*y*^. In cw-PL experiments, the samples were excited by the same laser beam as that used in the photocurrent measurements under the normal incident condition. The resulting PL emission from the GaAs substrate was collected in a backscattering geometry by a cooled Ge detector through a 0.8-m double grating monochromator. In the TR-PL experiments, a pulsed Ti-sapphire laser with a repetition rate of 76 MHz and a pulse duration of ∼2 ps was employed. Transient PL was detected by a streak camera in combination with a 0.5-m single grating monochromator. The time resolution of the whole TR-PL system is 2 ps.

### Data availability

All relevant data are available from the authors on request.

## Additional information

**How to cite this article:** Huang, Y. Q. *et al*. Spin injection and helicity control of surface spin photocurrent in a three dimensional topological insulator. *Nat. Commun.* **8,** 15401 doi: 10.1038/ncomms15401 (2017).

**Publisher's note:** Springer Nature remains neutral with regard to jurisdictional claims in published maps and institutional affiliations.

## Supplementary Material

Supplementary Information Supplementary Figures, Supplementary Notes and Supplementary References

## Figures and Tables

**Table 1:** Fitting parameters for the angular dependence of the helicity-dependent photocurrent.

**Sample**	**Excitation wavelength (nm)**	***C***_***x***_ **(nA·W**^**−1**^**)**	***C***_***y***_ **(nA·W**^**−1**^**)**	***D***_***x***_ **(nA·W**^**−1**^**)**	***D***_***y***_ **(nA·W**^**−1**^**)**
S1	800	−1.02±0.10	−0.06±0.08	0.13±0.07	0.39±0.06
S2	<813	−10.67±0.49	2.94	−0.45±0.25	−0.34±0.19
S2	≥813	−11.83±0.47	2.94	−0.57±0.24	−1.75±0.28

They were obtained by fitting the experimental data shown in Fig. 1e–g by equation (2). For Sample S2, the fitting parameter *C*_*y*_ is fixed and has the same value for both below (≥813 nm) and above (<813 nm) GaAs bandgap excitation as it arises from the same origin (a slight misalignment in the experimental geometry as discussed in the text). The uncertainty are received from the least-square-fitting procedure of the angular dependence data using equation (2).
