# Supplementary material for: Spin injection and helicity control of surface spin photocurrent in a three dimensional topological insulator
Source: Nat Commun. 2017 May 22;8:15401. doi: 10.1038/ncomms15401 (PMC5458147; doi:10.1038/ncomms15401)
Supplement: Supplementary Information — Supplementary Figures, Supplementary Notes and Supplementary References [file ncomms15401-s1.pdf]

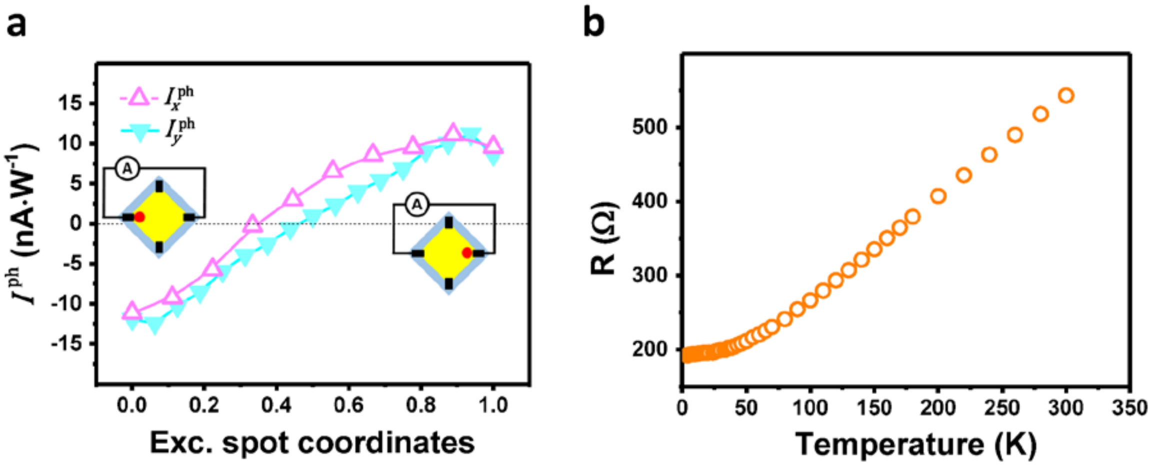

Supplementary Figure 1: Helicity-independent photocurrent. **a**, Helicity-independent photocurrent measured along the *x* (*y*) direction as a function of the *x* (*y*) coordinates of the laser spot. The coordinates are normalized so that they ranges from 0 to 1. **b**, Temperature dependence of resistance measured between the contact c1 and c3. All data were obtained from the S1 sample.

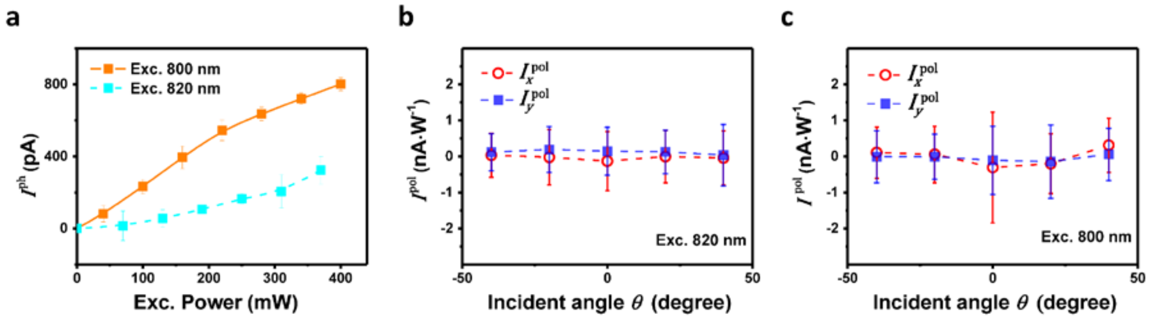

Supplementary Figure 2: **Photocurrent from the GaAs substrate. a**, Excitation power dependence of helicity-independent photocurrent measured on the bare GaAs (111)B substrate (i.e. Substrate 1) used in the growth of the studied Bi_2Te_3 thin film for the S1 sample, under the optical excitation below and above the GaAs bandgap (820 nm and 800 nm, respectively). **b** and **c** show the absence of helicity-dependent photocurrent in the same substrate under both excitation conditions. The error bars were estimated from the statistics of 300 data points collected in steady-state measurements.

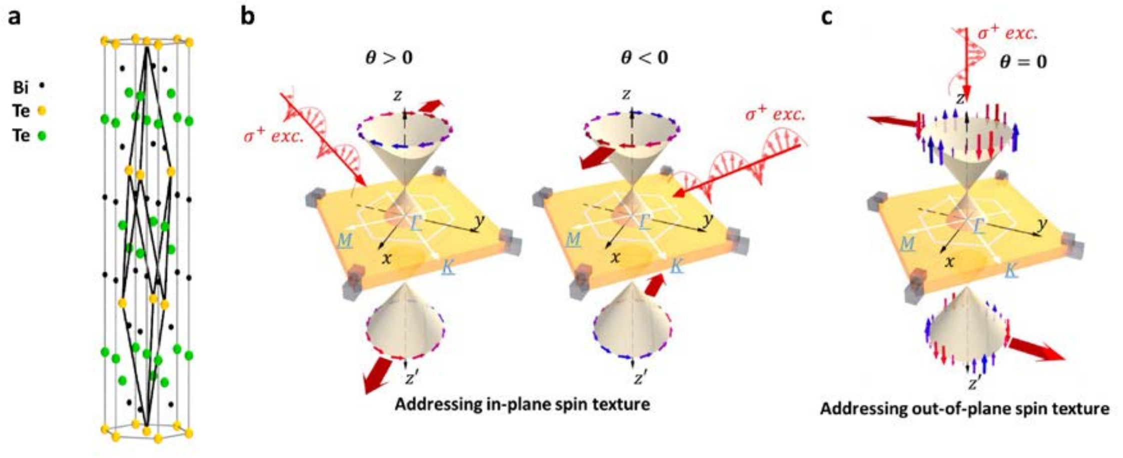

Supplementary Figure 3: Crystal structure and surface spin texture of of Bi_2Te_3. a, Crystal structure of Bi_2Te_3. **b,c,** Schematic illustrations of the spin texture orientations of the in-plane and the out-of-plane spin component for both top and bottom surfaces with inclined (**b**) and normal (**c**) incidence of the excitation laser beam. The corresponding spin current directions are highlighted by the thick red arrows.

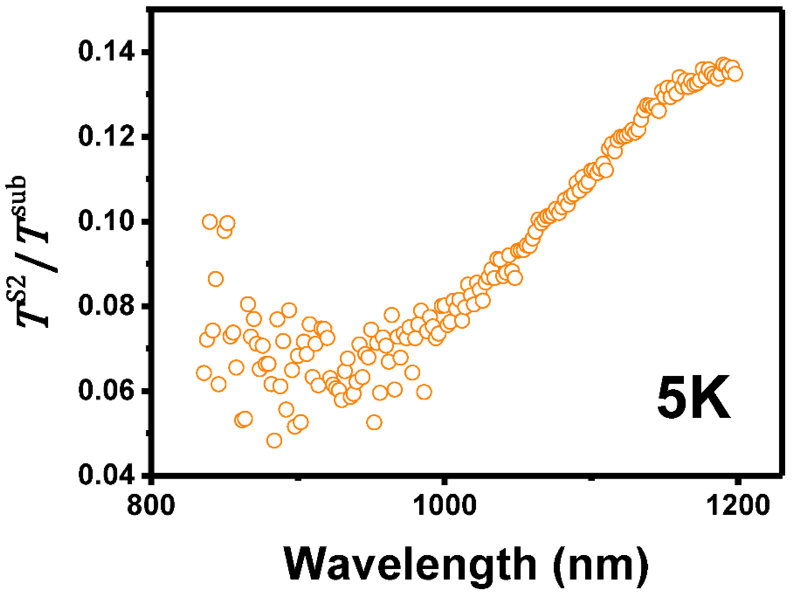

Supplementary Figure 4: Optical transmission. Optical transmission spectrum of the Bi_2Te_3 film obtained by dividing the transmission spectrum of the S2 sample (T^{S2}) by that from the bare GaAs substrate sample of Substrate 2 (T^{sub}).

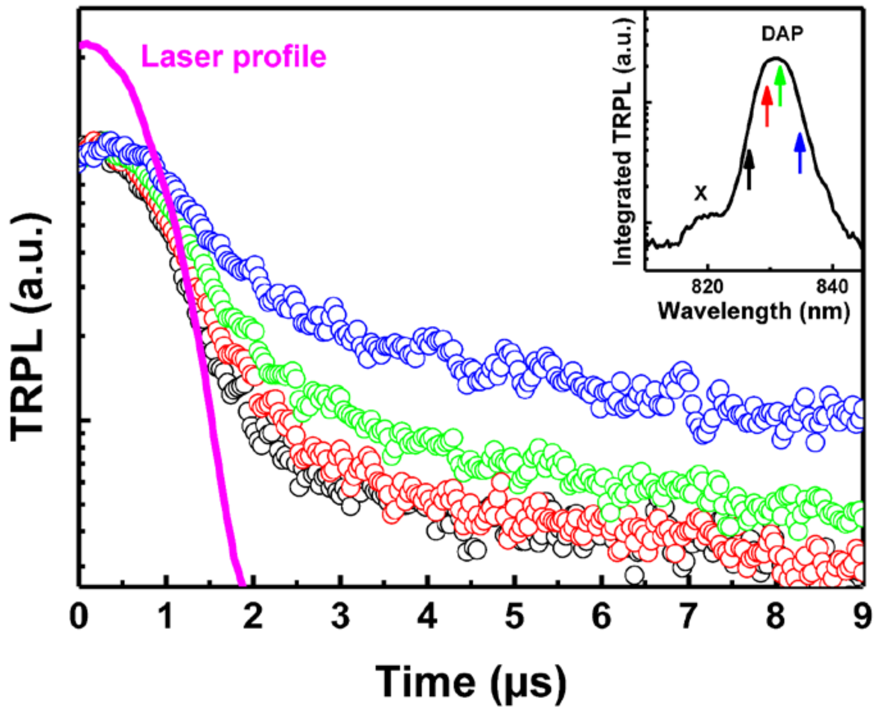

Supplementary Figure 5: Time-resolved photoluminescence. The normalized transient PL of the DAP emission measured at 5K from the bare substrate, Substrate 2 (the colored symbols), detected at the wavelengths marked by the arrows in the integrated PL spectrum shown in the inset. All the decay curves were taken under excitation with a photon energy above the GaAs bandgap. The solid line is the laser profile, which sets the time resolution of the transient PL measurements.

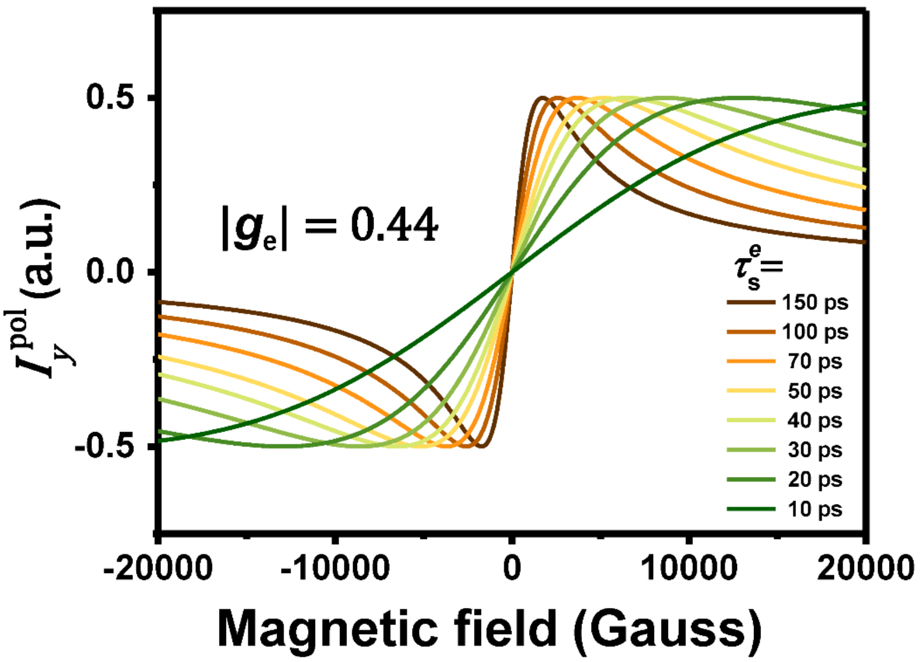

Supplementary Figure 6: Helicity-dependent surface photocurrent in a magnetic field. The simulated field dependence of I_y^{pol} as a function of τ_s^e, based on the Equation 3 of the manuscript.

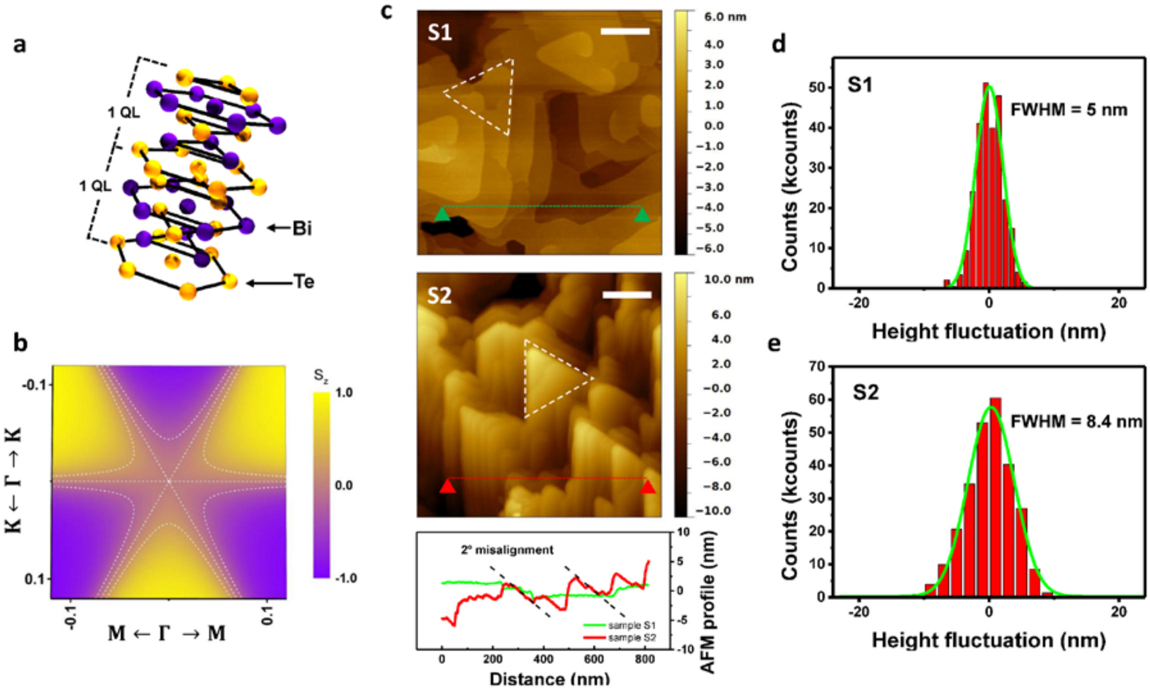

Supplementary Figure 7: Surface analysis. **a,** Illustration of the atomic structure of Bi_2Te_3 with the trigonal (001) surface plane. **b,** Illustration of the hexagonal warping effect by showing the out-of-plane spin texture for $|k_x, k_y| < 0.12$ Å^{-1}. Here, it can be clearly seen that the spin texture vanishes along the $\Gamma - \mathrm{M}$ direction. **c,** AFM images and the line scans of the height from the S1 and S2 samples. The dashed triangles outline the surface C_{3v} symmetry. The scale bar is 200 nm. The AFM profile is extracted from the showing AFM image. The 2° misalignment in S2 is highlighted. **d,** Statistical distributions of the surface height over the entire AFM image areas shown in (**c**).

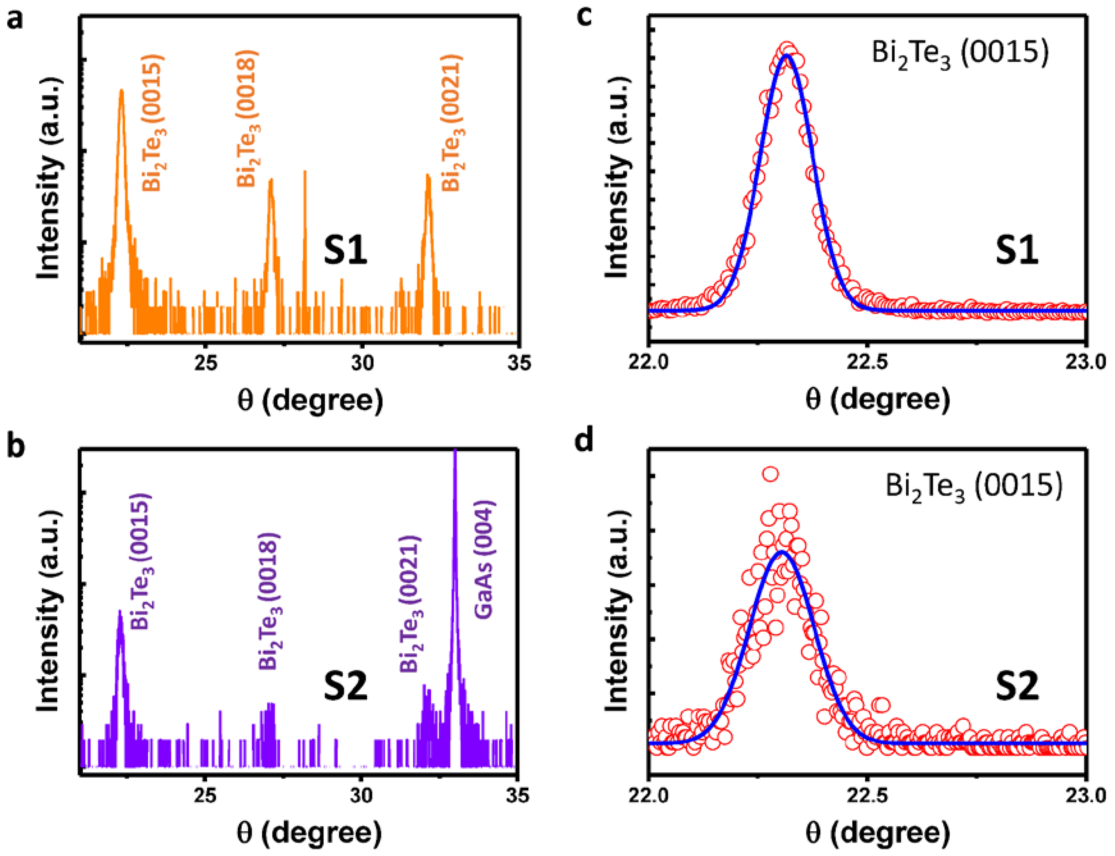

Supplementary Figure 8: XRD. **a,b,**The quintuple layer (QL) structure is identified by XRD $\theta - 2\theta$ scans of the sample S1 grown on a GaAs (111)B substrate and the sample S2 grown on a GaAs (100) 2° off-cut substrate. **c,d,**The XRD (0015) peaks of the two samples, where the symbols are the experimental data and solid lines are the fitting curves by a Gaussian lineshape.

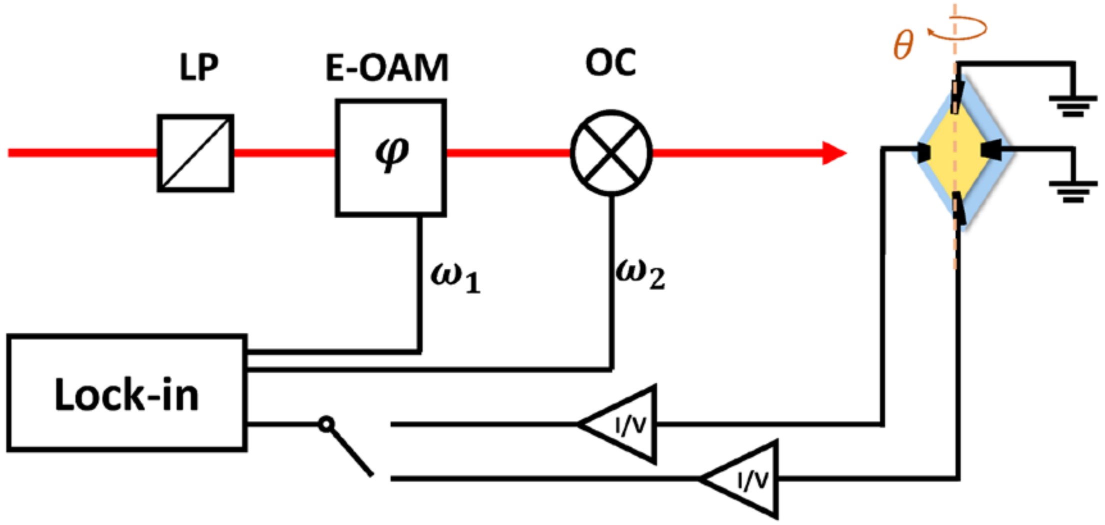

Supplementary Figure 9: Measurement setup. Schematic picture of the measurement setup, which allowed to selectively detect the helicity-dependent photocurrent by suppressing the irrelevant trivial effect.

Supplementary Note 1: Helicity-independent photocurrent

Helicity-independent photocurrent, denoted by I_x^{ph} (I_y^{ph}) for the current component along the *x* (*y*) direction, was measured under the zero bias condition. We found that the helicity-independent photocurrent depended on the excitation spot such that I_x^{ph} (I_y^{ph}) varied from a negative to a positive value when the laser spot was moved across the device along the *x* (*y*) coordinate as shown in Supplementary Figure 1(a). This behavior fits the description of bulk thermoelectric current created by imbalanced laser heating, which is a commonly observed effect in Bi_2X_3 (X stands for Se or Te) due to the high thermoelectric power in these materials [1-3]. This photo-thermoelectric current was shown to be associated with an electron-like behavior, propagating along the direction of the heat gradient. This is consistent with the intrinsic *n*-type metallic conduction as demonstrated by the results of temperature dependent resistance from the studied S1 sample shown in Supplementary Figure 1(b) and Hall measurements carried out using a standard Van der Pauw configuration (not shown here).

Supplementary Note 2: Control measurements of the GaAs substrates

To determine possible contributions of the GaAs substrates to the measured helicity-dependence photocurrent, we carried out careful measurements of photocurrent on the bare substrates alone under the same experimental conditions as that used in the studies of the TI. The results are shown in Supplementary Figure 2. Though helicity-independent photocurrent was generated, no helicity-dependent photocurrent could be observed under both above and below GaAs bandgap excitation. This result rules out any contribution of leakage current from the GaAs substrates in the sizable helicity-dependent photo-generated spin current observed in the TI devices.

Supplementary Note 3: Helicity-dependent photocurrent

The helicity-dependent photocurrent and its behavior are derived from a simple model following the relaxation time approximation [4]. To simplify calculations, here, we only consider the circular photo-galvanic effect (CPGE) by promoting electrons from a fully occupied Dirac cone, i.e. under the condition in which the Fermi level lies above the bulk conduction band edge (the effect of changing Fermi level will be address below in Supplementary Note 4). For the proposed scenario, helicity-dependent current is only carried by the optically created holes in the helical states while the electron counterpart created in the spin degenerated bulk states has a negligible contribution. Therefore, we can write the expression for the current arising from the CPGE by summing up the contributions from all helical states,

$$\mathbf{j} = \frac{2\pi e \tau_{\mathrm{p}}}{\hbar} \sum_{s,n,\boldsymbol{k}} \mathbf{v}\, |M(\mathbf{A},\mathbf{k})|^2 \delta\big(E^f(\mathbf{k}) - E^i(\mathbf{k}) - \hbar\omega\big), \qquad (1)$$

where τ_{p} is the hole momentum relaxation time, $\mathbf{v}$ describes the helical hole velocity and $M(\mathbf{A},\mathbf{k}) = \langle \varphi_s^f | \mathcal{H}_{\mathrm{int}} | \mathbf{k} \rangle$ is the optical transition matrix element between the helical initial state $|\mathbf{k}\rangle$ and the trivial finial state $|\varphi_s^f\rangle$ with the spin quantum number s. If we take a further assumption that $\mathbf{v}$ could be approximated as $n\hat{\mathbf{k}} \cdot v_{\mathrm{F}}$, where $n = \pm 1$ is the band index for the electron-/hole-like branch of the Dirac cone and v_{F} is the Fermi velocity at the Dirac point. $\delta\big(E^f(\mathbf{k}) - E^i(\mathbf{k}) - \hbar\omega\big)$ is inserted to ensure the energy conservation, which can be taken out in our simplified model.

To determine the photocurrent, we first need to work out the optical transition matrix. Using a $k \cdot p$ model, the effective Hamiltonian of the surface band can be expanded to the third order in $\mathbf{k}$,

$$\mathcal{H}_{\mathrm{surface}} = v_{\mathrm{F}}\big(k_x\sigma_y - k_y\sigma_x\big) + \frac{\lambda}{2}(k_+^3 + k_-^3)\sigma_z, \qquad (2)$$

where we eliminate the term that breaks the particle-hole symmetry and k-dependent correction to the Fermi velocity. Here $k_{\pm} = k_x \pm ik_y$ and λ characterize the hexagonal warping effect which has been shown to be important for Bi_2Te_3 from earlier theoretical and experimental ARPES studies [5-6]. We notice that the hexagonal warping term breaks the rotational symmetry and the helical states are now constrained by the bulk symmetry, say, the time reversal symmetry T and the lattice C_{3v} symmetry. The eigenstates of the Hamiltonian are expanded in the basis of the pseudospin up and down states $|\phi_s\rangle$ as $|\mathbf{k}\rangle = u_{\mathbf{k}}|\phi_\uparrow\rangle + v_{\mathbf{k}}|\phi_\downarrow\rangle$ and the spin texture is determined through the expansion coefficients,

$$\langle\sigma_x\rangle_{\mathbf{k}} = u_{\mathbf{k}}v_{\mathbf{k}}^* + u_{\mathbf{k}}^*v_{\mathbf{k}}\,, \langle\sigma_y\rangle_{\mathbf{k}} = iu_{\mathbf{k}}v_{\mathbf{k}}^* - iu_{\mathbf{k}}^*v_{\mathbf{k}}\,, \langle\sigma_z\rangle_{\mathbf{k}} = |u_{\mathbf{k}}|^2 - |v_{\mathbf{k}}|^2\,. \quad (3)$$

Under illumination, the optical transition is described by the interaction Hamiltonian of the general form $\mathcal{H}_{int} = -\frac{e}{c}\mathbf{A}\cdot\mathbf{v}$ with $\mathbf{A} = (A_x, A_y, A_z)$ being the Fourier transformation of light vector potential and $\mathbf{v} = (v_z, v_y, v_z)$ the velocity operator. Following a symmetry argument, the only non-vanishing matrix elements of the velocity operator are,

$$\langle\varphi_\uparrow^f|v_+|\phi_\downarrow\rangle = -\langle\varphi_\downarrow^f|v_-|\phi_\uparrow\rangle = i\alpha; \quad (4.1)$$

$$\langle\varphi_\uparrow^f|v_z|\phi_\uparrow\rangle = \langle\varphi_\downarrow^f|v_z|\phi_\downarrow\rangle = i\beta. \quad (4.2)$$

Here, $v_\pm = v_x \pm iv_y$ and α, β are the real value parameters that depend on the exact form of the interaction. This would immediately give the optical transition probability associated with an arbitrary helical state $|\mathbf{k}\rangle$,

$$\sum_s|M(\mathbf{A},\mathbf{k})|^2 = \frac{e^2}{4c^2}\Big\{\alpha^2\left(|A_x|^2 + |A_y|^2\right) + 2\alpha^2\Im\left(A_xA_y^*\right)\langle\sigma_z\rangle_{\mathbf{k}} + 4\alpha\beta\left[\Im(A_zA_x^*)\langle\sigma_y\rangle_{\mathbf{k}} - \Im\left(A_zA_y^*\right)\langle\sigma_x\rangle_{\mathbf{k}}\right]\Big\}. \quad (5)$$

Here, $\Im$ takes the imaginary part of the vector potential and the contributions from final states with different spin configurations are summed up. In our measurements, the excitation laser beam was fixed in the *y-z* plane with an incidence angle θ such that $\mathbf{A}_\pm = \frac{A}{\sqrt{2}}(\pm i, \cos\theta, \sin\theta)$ with $+/-$ sign corresponding to σ^+/σ^- excitation. The helicity-dependent photocurrent was

measured as the difference between σ^+ an σ^- excitation, which is only associated with the helicity-dependent part of the transition matrix $\Gamma_{\mathbf{k}}^{\mathrm{pol}} = \sum_s |M(\mathbf{A}_+, \mathbf{k})|^2 - |M(\mathbf{A}_-, \mathbf{k})|^2$. The equation for angular dependence used in this work can then be obtained as,

$$\Gamma_{\mathbf{k}}^{\mathrm{pol}} = \frac{e^2 A^2}{2c^2}\left(\alpha^2 \cos\theta \cdot \langle\sigma_z\rangle_{\mathbf{k}} - 2\alpha\beta \sin\theta \cdot \langle\sigma_y\rangle_{\mathbf{k}}\right). \tag{6}$$

We note that there also exists the helicity-independent transition matrix element, which reads

$$\Gamma_{\mathbf{k}}^{\mathrm{unpol}} = \sum_s |M(\mathbf{A}_+, \mathbf{k})|^2 + |M(\mathbf{A}_-, \mathbf{k})|^2 = \frac{e^2 A^2 \alpha^2}{4c^2}(1 + \cos^2\theta). \tag{7}$$

Following Supplementary Equation (7), one finds that $\Gamma_{\mathbf{k}}^{\mathrm{unpol}}$ will not contribute to any net current as it gives rise to the same hole population for both $|\mathbf{k}\rangle$ and $|-\mathbf{k}\rangle$ state.

Without the hexagonal warping effect (i.e. $\lambda = 0$), $|\mathbf{k}\rangle = \frac{1}{\sqrt{2}}(|\phi_\uparrow\rangle - nie^{i\theta_{\mathbf{k}}}|\phi_\downarrow\rangle)$ follows the dispersion of an ideal Dirac cone and $\langle\sigma_z\rangle_{\mathbf{k}}$ is found to be vanished for all $|\mathbf{k}\rangle$. This leads to a single-component helicity-dependent photocurrent density which reads,

$$\mathbf{j}_1 = \frac{2\pi\tau_{\mathrm{p}} v_{\mathrm{F}} e^3 A^2 \alpha\beta}{\hbar c^2} \hat{\mathbf{x}} \sin\theta \cdot \mathrm{N}. \tag{8}$$

Here, N is the total number of helical states that depend on the excitation area S_{exc}. Supplementary Equation (8) predicts a transverse photocurrent running along the *x* direction and having a nontrivial dependence on the incidence angle that gives a zero value under the normal incidence. Now we add the hexagonal warping effect by setting $\lambda \neq 0$. This immediately yields finite $\langle\sigma_z\rangle_{\mathbf{k}}$ and one would expect an addition current component from the first term in Supplementary Equation (6). Since $\langle\sigma_z\rangle_{\mathbf{k}}$ has only been experimentally observed for the electron-like band (n=1) of the Dirac cone, we limit our calculations to the upper helical band. We find that the photocurrent density has an amplitude depending on the electrical probing direction γ,

$$j_2(\gamma, \Delta) = \frac{\pi\tau_{\mathrm{p}} v_{\mathrm{F}} e^3 A^2 \alpha^2}{\hbar c^2} cos\theta \cdot \frac{S_{\mathrm{exc}}}{(2\pi)^2} \int_0^{k_{\max}} \int_{\gamma-\Delta}^{\gamma+\Delta} \frac{\cos 3\theta_{\mathbf{k}} \cos(\theta_{\mathbf{k}} - \gamma)}{\sqrt{\cos^2\theta_{\mathbf{k}} + \lambda^2 / v_{\mathrm{F}}^2 k^4}} k \cdot dk d\theta_{\mathbf{k}}. \tag{9}$$

Here, we restrict the current to that arising from small angles around the probing direction in k-space ($\gamma - \Delta < \theta_{\mathbf{k}} < \gamma + \Delta$) such that the integration in Supplementary Equation (9) does not vanish. We point out that this is a reasonable approach since the Dirac fermion has a suppressed probability for large scattering angles. As a result, photocurrent would primarily come from the states with their momenta along the electrical detection direction. Supplementary Equation (9) describes the second component of the helicity-dependent photocurrent, which originates from the out-of-plane spin texture and gives rise to the additional current component that does not vanish at $\theta = 0^{\circ}$.

We should note that our TI films are thin enough to allow light absorption by the bottom surface. Special care should therefore be exercised in terms of contributions from both top and bottom surfaces to the measured helicity-dependent surface spin photocurrent, especially considering that the two surfaces should exhibit opposite signs in the spin-momentum locking. Supplementary Figure 3 shows schematic illustrations of the orientations of the in-plane and out-of-plane spin texture components as well as their corresponding surface photocurrent directions for both top and bottom surfaces of the TI. Based on the symmetry of the Bi_2Te_3 crystal shown in Supplementary Figure 3(a), the spin texture and spin-momentum locking direction should be the same between the top and bottom surface in their own coordinates defined with their respective surface normal directions along z and z', but are opposite in the laboratory frame between the two surfaces for both in-plane and out-of-plane components of the surface spin texture as illustrated in Supplementary Figure 3(b,c). For example, the helicity of the in-plane spin texture on each surface can be visualized as being following a clockwise pattern around its corresponding surface normal axis. As the surface normal directions between the top and bottom surfaces are opposite in the laboratory frame, the helical surface state on one of the surfaces becomes counter-clockwise in a common laboratory frame. As a result, the same orientation of the in-plane spin texture

appears with the opposite sign of **k** between the top and bottom surfaces in the laboratory frame. Under the circularly polarized light excitation with a given finite incident angle θ in the *y*-*z* plane of the laboratory frame, which interacts with the TI surface state with a fixed spin orientation $\langle S_y \rangle$ in the laboratory frame, the direction of the helicity-dependent surface spin photocurrent I_x^{pol} is opposite between the top and bottom surfaces as illustrated in Supplementary Figure 3(b). The observation of a non-vanishing surface spin photocurrent thus requires a difference in the magnitude between the top and bottom surface current due to effects like light absorption or scattering in the bulk of the TI films, such that they are not completely canceled out. To obtain a quantitative estimate of the difference in light absorption between the top and bottom surfaces, we have performed optical transmission experiments on the studied Bi_2Te_3/GaAs samples and the bare GaAs substrate (to be presented in detail below in Supplementary Note 6). These transmission results show that only a few percent of the excitation light exits the bottom surface, meaning a significant attenuation of light passing through the Bi_2Te_3 film. Apart from light reflection at the two surfaces of the TI film, light absorption by the bulk of the film must have also contributed to the observed light attenuation judging from a sizable bulk photo-thermoelectric current detected in the imbalanced geometry shown in Fig.3a of the manuscript. From Fig.3a of the manuscript, the photocurrent generated from the light absorption by the TI film alone (with photon energy below the GaAs bandgap energy) is about 2.3 $nA\cdot W^{-1}$. The total photocurrent generated from the light absorption by both the TI film and GaAs substrate under the above GaAs bandgap excitation (e.g. at 1.65 eV) is about 3.3 $nA\cdot W^{-1}$. Assuming that the light absorption by the TI film is approximately wavelength independent within this narrow spectral range, which is well above the Bi_2Te_3 bandgap, the contributions from the TI film and the GaAs substrate to the total photocurrent are 2.3 and 1.0 $nA\cdot W^{-1}$, respectively. The light absorption by the TI film can be concluded to contribute to 70% of the total current, which is roughly 2 times higher than the contribution of

the photo-excited carrier injection from the GaAs substrate. The observed sizable light absorption by the bulk of the Bi_2Te_3 film results in an imbalance in light absorption between the top and bottom surfaces, thereby providing an explanation for the measurable helicity-dependent photocurrent in our study. We should note that the earlier work on helicity-dependent photocurrent reported in Supplementary Refs [1,7-8] was obtained in Bi_2Se_3 flakes of a thickness comparable to our Bi_2Te_3 films, where a similar imbalance in light absorption between the top and bottom surfaces must have provided the source for the measured helicity-dependent photocurrent.

Supplementary Note 4: Effect of changing Fermi level

Up to now, we only deal with the condition that the Fermi level is located well inside the bulk conduction band such that photocurrent can be derived by summing up the contributions from the holes created in the gap as the Dirac cone is originally filled. A major consequence of changing the Fermi level is that it effectively alters the available states for optical transitions. To demonstrate the influence of the Fermi level, we assume that the Fermi level is located in the bulk bandgap and try to derive the expression for $\mathrm{T} = 0$ K. Here we only focus on the out-of-plane spin-mediated photocurrent j_2, which is the one that we have observed the clear feature due to the excitation across the GaAs bandgap. In this scenario, the filled helical states below the Fermi level allows for optical transition from the gap state $|\mathbf{k}\rangle$ to higher lying conduction band states $\left|\varphi_s^f\right\rangle$ and generating hole current, while the empty states $|\mathbf{k}'\rangle$ above the Fermi level would accept electrons promoted from the valence band states $\left|\varphi_s^i\right\rangle$ and give rise to electron current. The hole current can be obtained directly from Supplementary Equation (9) with $k_{\max}$ replaced by the Fermi wave vector k_{F}. By replacing $|\mathbf{k}\rangle$ and $\left|\varphi_s^f\right\rangle$ by $|\mathbf{k}'\rangle$ and $\left|\varphi_s^i\right\rangle$ in Supplementary Equation (4) and applying few modifications, we obtain the corresponding matrix elements for calculating electron current,

$$\langle\phi_\uparrow|v_+|\varphi_\uparrow^i\rangle = -\langle\varphi_\downarrow^f|v_-|\phi_\uparrow\rangle = i\alpha'; \tag{10.1}$$

$$-\langle\phi_\downarrow|v_z|\varphi_\downarrow^i\rangle = -\langle\phi_\uparrow|v_z|\varphi_\uparrow^i\rangle = i\beta'. \tag{10.2}$$

Here, α' and β' are another set of real valued parameters, which are not necessarily equivalent to α and β. Under the normal incidence $\mathbf{A}_\pm = \frac{A}{\sqrt{2}}(\pm i, 1{,}0)$, compared to Supplementary Equation (6), the polarization dependent transition probability for the out-of-plane spin texture now contains a negative sign,

$$\Gamma_{\mathbf{k}'}^{\mathrm{pol}} = -\frac{e^2A^2}{2c^2}\alpha'^2 \cdot \langle\sigma_z\rangle_{\mathbf{k}'}\,. \tag{11}$$

This result is a direct consequence of a changed matrix form in Supplementary Equation (10) that v_+ (v_-) is now associated with the pseudospin up (down) state, which is the exact reversal of Supplementary Equation (4). Since the electron carries a negative charge, the combined effect is that electron current is also described by Supplementary Equation (9) with α'^2 substituting α^2 and integration over k_F to k_max. This result essentially shows that regardless the change of the Fermi level, helicity-dependent photocurrent maintains its polarity even though the magnitude of the current might vary. This excludes a possible contribution from a change of the Fermi level upon carrier injection from GaAs, at least not to the degree that I_y^pol reverses its polarity between excitation above and below the GaAs bandgap. The observed excitation wavelength dependent feature of I_y^pol can then be unambiguously attributed to the spin injection from GaAs.

Supplementary Note 5: Optical transmission

To estimate the extent of the light penetration through the TI film, we carried out optical transmission measurements of both sample S2 and the bare GaAs substrate. The optical transmission spectrum for the TI film is shown in Supplementary Figure 4, obtained by dividing the transmission spectrum of the S2 sample (T^{S2}) by that of the GaAs substrate (T^{sub}). The observed strong attenuation of light by the TI film is contributed by both light reflection at the two surfaces and light absorption by the bulk of the TI film. Data above the GaAs bandgap is not available as the light is completely absorbed by the GaAs substrate.

Supplementary Note 6: Photoluminescence polarization of the donor-acceptor pair recombination

First of all, we should point out that the donor-acceptor pair (DAP) photoluminescence (PL) emission arises from the recombination between electrons localized at donor sites and holes localized at acceptor sites, which do not contribute to spin injection to the TI. They are totally different from the free carriers and free excitons that are mobile and responsible for the spin injection, and in no way reflect spin polarization of the latter. In fact, the DAP emission from GaAs is often found unpolarized regardless of the polarization of conduction band (CB) electrons [9,10]. This is due to an extremely long lifetime of the electrons (holes) localized at the donors (acceptors) involved in distant DAP recombination [11] that is much longer than its typical spin relaxation time, resulting in zero circular polarization degree [9,10]. The effect of lifetime on spin polarization is well understood in a semiconductor and can be described by the well-known relationship $\rho = \rho_0/(1 + \tau/\tau_s)$ [12], where ρ_0 and ρ are the spin polarization degrees of the concerned carriers or excitons before and after undergoing spin relaxation, respectively. τ and τ_s are the lifetime and the spin relaxation time. The long lifetimes of the electrons (holes) localized at the donors (acceptors) were confirmed in our samples by a slow decay of the DAP emission measured at 5 K from the bare substrate sample, Substrate 2, as shown in Supplementary Figure 5, with a decay time substantially longer than 1 μs for the distant pairs at the low energy side of the emission. During such a long lifetime, any initial spin polarization preserved during capture of free CB electrons (valence band holes) to the donors (acceptors) is lost due to the faster spin relaxation processes that are further promoted by the interaction between the recombining localized electron-hole pairs. It is therefore not surprising that circular polarization was not observed for the distant DAP emission in our samples.

We should note that, for the bare substrates under a high excitation density, the high-energy side of the DAP emission starts to develop a finite PL polarization that increases with increasing emission energy as shown in Fig.2(a,b) of the manuscript. This is because the PL emission within this spectral range arises from recombination between close donor-acceptor pairs with a shorter decay time [13], as the DAP decay time τ follows an exponential function of the pair distance R by the relation $1/\tau \propto \exp(-2R/a_0)$ where a_0 denotes the larger Bohr radius of the donors and acceptors. When the localized electron/hole lifetime becomes shorter than their spin relaxation time τ_s, PL polarization $P^{\mathrm{PL}} = P_0^{\mathrm{PL}}/(1 + \tau/\tau_s)$ is expected to develop to a finite value. Within the same spectral range, the free-to-bound (FB) emission that arises from the recombination between CB electrons and holes localized at the acceptors may also contribute under the strong excitation condition.

Supplementary Note 7: Magnetic field dependence of helicity-dependent photocurrent

In the field dependent experiments, we measured helicity dependent photocurrent in a steady state under a non-equilibrium condition (i.e. under the cw optical excitation). Here, the electrons with their initial spin orientation along the *z* direction (defined by optical pumping under the normal incident condition) are continuously generated in GaAs by circularly polarized light excitation with $E_{\mathrm{exc}} > E_g^{\mathrm{GaAs}}$. In a transverse magnetic field along the *y* direction, the electron spin undergoes a Larmor precession in the *x*-*z* plane at the Larmor frequency $\Omega = g_e \mu_{\mathrm{B}} B/\hbar$. Such precession can be interrupted by events of electron spin relaxation and electron losses (due to injection, trapping, recombination, etc.), which is commonly described by an effective electron spin lifetime τ_s^e. It is a dynamic balance between these two fast processes that results in a finite value in an averaged non-equilibrium spin projection along the x direction, $\langle S_x \rangle$, and thus measurable I_y^{pol}. The magnitude of $\langle S_x \rangle$ is determined by the relative values of τ_s^e and the spin precession period $(1/\Omega)$, which should be field dependent. This is in fact in the same spirit of the well-known Hanle effect, in which spin lifetime can be determined by measuring the steady-state value of $\langle S_z \rangle$ in a transverse magnetic field. (A quantitative understanding of the precession process and its field dependence can be found in Supplementary Refs.14-17, which are now widely used to prove spin transport and to measure the spin relaxation time in graphene, TI and etc.) The general trend of the field dependence can be visualized as follows. At zero field, the driving force (i.e. the transverse magnetic field) to tilt the spin axis away from the *z* axis towards a particular in-plane direction is absent. Therefore, $\langle S_x \rangle$ is expected to be zero and does not lead to I_y^{pol}. With increasing magnetic field, $\langle S_x \rangle$ starts to develop along either +*x* or –*x* direction depending on the direction of the field. This increase in $|\langle S_x \rangle|$ and thus $\left| I_y^{\mathrm{pol}} \right|$ continues until reaching the high-field range ($\tau_s^e \Omega > 1$) when the spin precession becomes fast enough to undergo a number of precession cycles within the electron spin lifetime, causing the average

$|\langle S_x \rangle|$ to decrease and finally approaches zero at an extremely high field when $\tau_s^e \Omega \gg 1$. The field range where $|\langle S_x \rangle|$ and $\left|I_y^{\mathrm{pol}}\right|$ reach their maximum values depends on τ_s^e, as illustrated by the simulated field dependence of I_y^{pol} shown in Supplementary Figure 6 based on the Eq.(3) of the manuscript. Due to a small value of τ_s^e, the experimentally measured $\left|I_y^{\mathrm{pol}}\right|$ in our samples just reaches its maximum value within the applied field range (-0.5T to +0.5T) shown in Fig.5a of the manuscript. Over a wider field range up to 1T as shown in Fig.5c,d of the manuscript, a hint of $\left|I_y^{\mathrm{pol}}\right|$ starting to decline can be seen at an absolute field above 0.8T.

We should point out that τ_s^e deduced from the fitting is an effective averaged value, as the experimental results were obtained from ensemble electrons in GaAs with a distribution of τ_s^e depending on their proximity to the Bi_2Te_3/GaAs interface. Such a distribution of τ_s^e could provide an explanation for the observed broadening of the experimental curves at high fields, as the simulations in Supplementary Figure 6 suggest.

Supplementary Note 8: Materials and crystal orientations

The Bi_2Te_3 thin films were grown on the GaAs (111)B and (100) 2° off-cut substrate, labeled as S1 and S2 respectively, using the technique of molecular beam epitaxy. From X-ray diffraction (XRD) and atomic force microscopy (AFM), the primary crystallographic orientations of the as-grown S1 and S2 films were identified and correlated with the *x* and *y* directions defined in the photocurrent measurements. As illustrated in Supplementary Figure 7(a), the Bi_2Te_3 films are layer structures with surface symmetry of C_{3v}, which includes a three-fold rotation symmetry and a vertical mirror plane. This can be explicitly seen from the AFM images in Supplementary Figure 7(c) where atomic layer fluctuations are shown to have a trigonal shape adapted to the symmetry. We note, from the AFM images, that a majority of the trigonal shaped islands are aligned with the orientation highlighted by the dashed triangle. The mirror plane of the triangle is rotated away from the diagonal axes of the samples, whereas the *x* and *y* directions in the photocurrent measurement geometry coincide with the diagonal axes of the samples. We point out that this is a necessary requirement to observe any spin-mediated current originating from the hexagonal effect, since the crystallographic direction correlated with the mirror plane belongs to the *k*-point along the $\Gamma - \mathrm{M}$ direction in the reciprocal space where the out-of-plane spin texture vanishes [see Supplementary Figure 7(b)].

To obtain a more reliable estimate of the surface height fluctuation, we have performed a quantitative, statistical analysis of the AFM images. The full-width-at-half-maximum (FWHM) values calculated from the statistical distributions over the 1 μm × 1 μm area of the AFM images are 5 nm and 8.4 nm for the sample S1 and S2, respectively, see Supplementary Figure 7(d,e). The observation of non-vanishing surface spin photocurrent over a long distance on the order of mm in these two samples in fact shows that the surface spin effect is rather tolerant to TI film thickness variations (up to 10-15% known in our samples).

The XRD measurements were performed with a typical $\theta - 2\theta$ scan for both S1 and S2 samples, and the result are shown in Supplementary Figure 8. For both samples, after removal of the substrate peak, the diffraction peaks are found to be the same and are originated only from the planes parallel to the trigonal lattice Bi_2Te_3 (001). This shows that, regardless of the crystallographic orientations of the substrates, the growth of Bi_2Te_3 took place along its trigonal [001] direction resulting in a layered structure with the (001) surface.

In principle, the XRD peak width could to some degree reflect the grain size, if it is a dominant factor limiting the XRD linewidth. Empirically, this can be estimated by using the Scherrer equation, which yields crystallites with a dimension of 30 and 24 nm for sample S1 and S2 based on their $\Delta(2\theta)$ values of 4.9×10^{-3} and 6.1×10^{-3} rad extracted from the Gaussian line fitting of the Bi_2Te_3(0015) peaks [see Supplementary Figure 8(c,d)]. However, this estimation does not account for other factors, like film thickness, instrumental broadening, strain distribution, lattice imperfection etc., some of which become critically important in thin film samples. In fact, it has been shown by J. Park *et al.* that the XRD peak broadening in the typical $\theta - 2\theta$ scan of a Bi_2Te_3 thin film grown on a Si substrate was actually limited by the film thickness [18]. The estimated values of the crystallite sizes from the analysis based on the Scherrer equation could very likely be mainly limited by the film thickness and its associated fluctuations in our samples, but not by the grain sizes. This is consistent with the AFM studies, see, e.g., Supplementary Figure 7(c), which show domain sizes to be much larger than 50 nm.

Supplementary Note 9: Measurement techniques

The lock-in technique was used to selectively detect the photocurrent of interest. To do so, we guided a wavelength-tunable Ti-sapphire laser beam through a linear polarizer (LP) in conjunction with an electro-optic amplitude modulator (E-OAM). With well-defined linear polarized light as an input, the E-OAM decomposed the light into two orthogonally polarized light and allows to control the exact polarization by controlling the phase shift between the two components. We used a triangle wave to modulate the excitation polarization with a periodic sequence following $\sigma^+ \rightarrow \sigma^y \rightarrow \sigma^- \rightarrow \sigma^y \rightarrow \sigma^+ \ldots$ at a modulation frequency ω_1. We could also modulate the excitation light intensity using an optical chopper (OC) at a different frequency ω_2 as showed in Supplementary Figure 9. In principle, by employing both polarization and intensity modulations, we could simultaneously identify helicity-dependent (I^{pol}) and helicity-independent (I^{ph}) current that were separated in the frequency space. However, in order to eliminate any unwanted effect from parasitic capacitance at high frequencies, both ω_1 and ω_2 were chosen to be low and closed to 170 Hz. Therefore, I^{pol} and I^{ph} were measured one-by-one but with the same optical alignment. During the measurements of I^{pol}, especially in the angular dependent measurements when the sample needed to be rotated with respect to the excitation laser beam, the excitation spots was maintained by moving the laser spots across the sample until a point (vanishing point) was found where the helicity-independent I^{ph} along both *x* and *y* directions were vanished. This was also an important step to filter out any artifacts that were unrelated to helicity but were accompanied by a change in the incident angle of the light beam. After the vanishing point was reached, the optical chopper was kept in a constant open state and I^{pol} was measured independently in phase with ω_1. We should note that both I^{pol} and I^{ph} were measured directly from the devices without applying electric bias. A current preamplifier was used to magnify the current and to convert the current signal to a sizable voltage signal, which was subsequently detected

by the lock-in amplifier. A schematic picture of the experimental setup is illustrated in Supplementary Figure 9.

Supplementary References

[1] Yan, Y. *et al.* Topological surface state enhanced photothermoelectric effect in Bi_2Se_3 nanoribbons. *Nano Lett.* **14,** 4389–94 (2014).

[2] DiSalvo, F. J. Thermoelectric Cooling and Power Generation. *Science* **285,** 703–6 (1999).

[3] Gabor, N. M. *et al.* Hot carrier-assisted intrinsic photoresponse in graphene. *Science* **334,** 648–52 (2011).

[4] Junck, A., Refael, G. & Von Oppen, F. Photocurrent response of topological insulator surface states. *Phys. Rev. B* **88,** 075144 (2013).

[5] Fu, L. Hexagonal warping effects in the surface states of the topological insulator Bi_2Te_3. *Phys. Rev. Lett.* **103,** 266801 (2009).

[6] Wang, Y. H. *et al.* Observation of a warped helical spin texture in Bi_2Se_3 from circular dichroism angle-resolved photoemission spectroscopy. *Phys. Rev. Lett.* **107,** 207602 (2011).

[7] McIver, J. W., Hsieh, D., Steinberg, H., Jarillo-Herrero, P. & Gedik, N. Control over topological insulator photocurrents with light polarization. *Nat. Nanotechnol.* **7,** 96–100 (2011).

[8] Kastl, C., Karnetzky, C., Karl, H. & Holleitner, A. W. Ultrafast helicity control of surface currents in topological insulators with near-unity fidelity. *Nat. Commun.* **6,** 6617 (2015).

[9] Miller, R. C., Tsang, W. T. & Nordland, W. A. Spin-dependent recombination in GaAs. *Phys. Rev. B* **21**, 1569–75 (1980).

[10] Furthmeier, S. et al. Enhanced spin-orbit coupling in core/shell nanowires. *Nat. Commun.* **7**, 12413 (2016).

[11] Williams, F. Donor-acceptor pairs in semiconductors. *Phys. Stat. sol.* **25**, 493-512

(1968).

[12] Meier, F. & Zakharchenya, B. P. Optical Orientation (North-Holland, 1984).

[13] Park, J. *et al.* Crystal structure and epitaxy of Bi_2Te_3 films grown on Si. *Appl. Phys. Lett.* **101**, 221910 (2012).

[14] Ganichev, S. D. & Prettl, W. Spin photocurrents in quantum wells. *Journal of Physics: Condensed Matter* **15**, R935–R983 (2003).

[15] Belkov, V. V & Ganichev, S. D. Magneto-gyrotropic effects in semiconductor quantum wells. *Semicond. Sci. Technol.* **23**, 114003 (2008).

[16] Tombros, N., Jozsa, C., Popinciuc, M., Jonkman, H. T. & van Wees, B. J. Electronic spin transport and spin precession in single graphene layers at room temperature. *Nature* **448**, 571–574 (2007).

[17] Vaklinova, K., Hoyer, A., Burghard, M. & Kern, K. Current-Induced Spin Polarization in Topological Insulator-Graphene Heterostructures. *Nano Lett.* **16**, 2595–2602 (2016).
